# Synthesis, Characterization, and *In Vitro* and *In Vivo* Evaluations of Cellulose Hydrogels Enriched with *Larrea tridentata* for Regenerative Applications

**DOI:** 10.1155/2020/1425402

**Published:** 2020-04-21

**Authors:** Karla Lizette Tovar-Carrillo, Rosa A. Saucedo-Acuña, Judith Ríos-Arana, Genaro Tamayo, Dalia Abril Guzmán-Gastellum, Beatriz A. Díaz-Torres, Salvador David Nava-Martínez, León Francisco Espinosa-Cristóbal, Juan Carlos Cuevas-González

**Affiliations:** Institute of Biomedical Sciences, Autonomous University of Ciudad Juarez, Juarez City, Chihuahua, Mexico

## Abstract

**Introduction:**

Tissue engineering is an elementary necessity for several applications in the biomedical field through the use of several biopolymers derived from plants. *Larrea tridentata* (LT) is a very used plant for various medicinal applications with interesting properties; however, its use into cellulose hydrogels for possible regenerative therapeutics is still limited. Cellulose films could be applied in medical field as wound healing, scaffold for connective tissue for periodontal applications, and so on. The aim of this study was to evaluate the mechanical properties and *in vivo* and *in vitro* biocompatibility of cellulose hydrogels that have been enriched with LT in a rat model.

**Methods:**

By *in vivo* and *in vitro* assays, the concentration of LT was varied from 1 to 5 wt%, respectively. Hydrogel films were implanted intramuscularly into female Wistar rats, 250 g in weight and aged 2 months, to analyze their cytocompatibility and biocompatibility.

**Results:**

No case showed any evidence of inflammation or toxicity. Regarding cell morphology and adhesion, the prepared LT cellulose films had better cytocompatibility values than when polystyrene (PS) dishes were used as the control. In all cases, the results suggest that the addition of LT to the hydrogel films did not affect their cytocompatibility or biocompatibility properties and increases their clinical application due to the reported uses of LT.

**Conclusions:**

Cellulose hydrogel films enriched with LT have the potential to be used in the biomedical field acting as regenerative scaffolds.

## 1. Introduction

In recent years, several approaches have been used to design suitable scaffolds for tissue regeneration [1, 2]. Tissue regeneration is an important discipline in understanding the development of an organ and for several applications, such as in medicine, for repair, treatment, wound healing, and function [3–5]. For this purpose, the scaffold should mimic the extracellular matrix as much as possible to provide a suitable environment for cell adhesion and, subsequently, tissue regeneration. However, it has been difficult to establish the ideal biocompatible scaffold to maintain long-term cell proliferation and promote tissue regeneration. One strategy in improving biocompatibility is to use natural polymers, such as collagen [6], hyaluronic acid [7], alginate [8], and chitosan [9], for the development of hydrogels. Among them, cellulose is the most common biopolymer, consisting of *β*-glucose, and is the most abundant material in nature [1, 2]. Moreover, hydrogels are favorable candidates for biological application and as a biocompatible material, due to their nontoxic properties and high water content and softness, which are consistent with the environment of natural tissues [3–5].

Further, cellulose hydrogels undergo good biointegration with surrounding tissues [10–12], allowing cell adhesion and tissue growth with nonadverse reactions to the material. *Larrea tridentata* (LT) is known as creosote bush and greasewood as a plant used as a medicinal herb. This shrubby plant belongs to the family Zygophyllaceae and predominates in deserts in the southwest United States and central and northern Mexico [12]. In addition, it is an important plant with a long history of medicinal use [13]. *L. tridentata* contains a series of lignans, flavonoids, condensed tannins, triterpene saponins, and naphtha quinones with antimicrobial, antioxidant, and antitumor activities [14]. Several studies have reported its ability to rejuvenate injured skin, wounds, and burned skin [12–15].

In this study, LT cellulose hydrogel films were prepared from agave bagasse waste [16–18]. Our group has been examining cellulose hydrogels that have been prepared from various biomasses, including sugarcane and bamboo fibers, for the fabrication of hydrogels that have high cytocompatibility and facilitate cell propagation [19, 20]. Few studies have addressed the biointegration of cellulose hydrogels *in vivo*, and none has incorporated LT for its healing properties. Necessity of nontoxic and biocompatible scaffolds rises with potential medical applications such as wound healing for diabetic patients and connective tissue regeneration for periodontal applications before and after dental treatments. Consequently, we focused on evaluation of cell morphology, adhesion, and cyto- and biocompatible properties of LT cellulose hydrogels in Wistar rats. Although a few attempts have been made to analyze hydrogel properties, studies on cell adhesion, morphology, and cyto- and biocompatible properties for tissue regeneration are still less in biomedical applications. On the basis of our previous work [10, 11, 16], the research of LT cellulose hydrogels could apply as a sustainable material for medical applications. As a frontier study, the present work can provide important evidence on tissue regeneration.

## 2. Materials and Methods

### 2.1. Collection of Samples

Tequilana Webber bagasse was obtained from Corralejo Tequila Company, Mexico. NN-Dimethylethylenediamine (DMAc), lithium chloride, ethanol, sodium hydroxide, sodium hypochlorite, sulfuric acid, the bicinchoninic acid (BCA) kit, fetal bovine serum (FBS), and bovine serum albumin (BSA) were purchased from Sigma-Aldrich. Phosphate-buffered saline (PBS, Dulbecco Co., Ltd.), trypsin-EDTA, and formaldehyde (37 vol% aqueous solution) were purchased from Chemical Tech Co., Ltd. For the cell culture experiments, NIH3T3 mouse embryonic fibroblasts were purchased from Invitrogen (Japan).

### 2.2. LT Cellulose Hydrogels

For the preparation of agave cellulose solutions, agave bagasse fibers underwent chemical treatment per our previous report [10]. Then, the treated agave fibers were used to prepare cellulose solution by solvent exchange with DMAc/LiCl [19]. To obtain LT cellulose hydrogels, dried LT was incorporated into the solvent exchange system. LT content was varied from 1 to 5 wt%. Transparent films were obtained by the phase inversion method by pouring 10 g of LT cellulose solution onto a glass tray in an alcohol atmosphere overnight. Cellulose films were washed with ethanol 3 times to remove traces of solvent in a shaking bath for 36 h. Then, the films were stored in PBS at 4°C.

### 2.3. Characterization of Hydrogels

To evaluate the mechanical properties of the hydrogel films, tensile strength and elongation were measured using an LTS-500N-520 universal testing machine with 2.5 kNcel. Hydrogel samples (1 mm × 10 mm × 50 mm) were cut and uniaxially deformed along their longest axis [20]. In 1 experiment, 5 samples were measured for each mimosa content. In total, 3 experiments were performed. Tensile strength (N/mm^2^) and elongation (%) were calculated per the following equations:

(1) $$\begin{array}{c} \text{tensile strength }\left(\text{N}/{\text{mm}}^{2}\right)=\frac{\text{maximum load}}{\text{cross}‐\text{sectional area}},\\ \text{elongation }\left(\%\right)=\frac{\left(\text{final length}\left(\text{mm}\right)\right)–\left(\text{initial length}\left(\text{mm}\right)\right)}{\left(\text{initial length}\left(\text{mm}\right)\right)}\times 100. \end{array}$$

Water swelling of the hydrogel films was evaluated by measuring the equilibrium water content (EWC). Weights of dried and hydrated samples were registered as follows [21]. The weight of dried hydrogel samples (5 mm × 5 mm) was determined, after which the samples were immersed in distilled water for 36 h. Finally, the samples were removed, and the weight of the hydrated film samples was calculated.

(2) $$\begin{array}{c} \text{EWC}=\left[\frac{\left(W_{\text{s}}–W_{\text{d}}\right)}{W_{\text{s}}}\right]\times 100, \end{array}$$

where *W*_s_ is the weight of the hydrated samples and *W*_d_ is the dry weight of the sample [20, 21]. Five samples were used for each type of mimosa cellulose film for each experiment. Six experiments were performed to calculate the EWC.

The contact angle was evaluated using a goniometer. Three film samples (20 mm × 20 mm) were tested for each LT hydrogel [20]. The hydrogel sample was adhered to a glass surface and placed in the goniometer. Then, 3 mL distilled water was dropped onto the material, and the angle that formed between the water drop and the surface of the sample was calculated. For each experiment, 3 samples were used for each hydrogel sample. The data of 5 experiments were averaged.

FT-IR spectroscopy was used to examine chemical functional groups in dry LT cellulose hydrogel samples using FT-IR 4100 series (JASCO Corp., Japan). Thin hydrogel film having about 20 *μ*m of thickness was placed between CaF_2_ windows (30 mm diameter, thickness 2 mm, Pier Optics Co., Ltd.). For measurement of scanning electronic microscopy (SEM), after the hydrogel film sample was dried, samples were coated with a gold layer. The SEM images were recorded using JSM-5310LVB (JEOL, Japan).

### 2.4. Protein Adsorption

Protein adsorption by the LT hydrogel films was measured by bicinchoninic acid assay [22]. Hydrogel films (5 mm × 5 mm) were immersed in 1 mL PBS and FBS that contained 1 mg/mL, respectively, and incubated for 4 h at 37°C. Samples were placed in tubes with 1 mL Dulbecco's modified Eagle's medium (DMEM) that was enriched with 10 vol% FBS and incubated for 4 h at 37°C. After the incubation, the samples were rinsed 3 times for 10 min in PBS to remove excess proteins. Then, 2 mL sodium dodecyl sulfate (SDS) 2 wt% solution was added to each sample and placed in a shaking bath at 25°C. The amount of adsorbed protein was measured at 562 nm against a curve that was obtained from pure protein samples [19]. For each experiment, 3 samples were used for each hydrogel, and the data for 6 experiments were analyzed and averaged.

### 2.5. Cell Culture

NIH3T3 mouse embryonic cells were used for the measurements of cell viability and morphology assays [20]. Circular hydrogel samples (30 mm in diameter) were sterilized with 70% ethanol for 30 min before the experiments. Cell dispersion solution was prepared in DMEM with a cell density of 8 × 10^3^ cm^−2^. Then, 2 mL dispersion solution was added to each sample and incubated at 37°C in a CO_2_ atmosphere. Samples were analyzed after 4, 24, 48, and 72 h in culture with regard to the number of adherent cells, and cell morphology was examined. Four samples were evaluated for each mimosa hydrogel for each time point. The experiments were tested statically by one-way analysis of variance (ANOVA), followed by Student's *t*-test, and the significance level was *p* < 0.05.

### 2.6. Cell Morphology

The cytotoxicity of adherent cells on the hydrogel surface was evaluated under an inverse microscope (Olympus CKX4) in samples at various cell culture times (4, 24, 48, and 72 h) [10]. After the culture, the PS dish that contained the hydrogel sample was washed twice with 2 mL PBS. Then, 2 mL 3.7% formaldehyde was used to fix the adherent cells to the hydrogel surface. The area of each was divided into four sections, each of which was analyzed separately under the microscope. Twenty images were taken of each section and analyzed in cellSens to calculate morphological parameters, such as the aspect ratio, cell area, and long axis. The results were based on 10 independent experiments.

### 2.7. *In Vivo* Assay

The *in vivo* assay was performed by intramuscular implantation of hydrogel film in rats. Female Wistar rats, weighing 250 g and aged 2 months, were used for the assay [11]. In all of the experiments, the rats were treated before and after surgery under the guidelines of the Animal Ethics Committee of the Autonomous University of Ciudad Juarez (approval number CIBE-2017-1-45). Before surgery, hydrogel samples (5 mm × 5 mm), 0.82 to 1.21 mm thick, were immersed in a shaking bath for 36 h to eliminate traces of solvent. Then, they were sterilized with 70% ethanol for 5 minutes and washed twice with PBS and stored. Eighteen rats were divided randomly into 6 groups for each experiment, including a control group. Four experiments were performed, varying the lifetime of the rats. Before surgery, the rats were weighed and then anesthetized using isoflurane.

**Figure 1:**
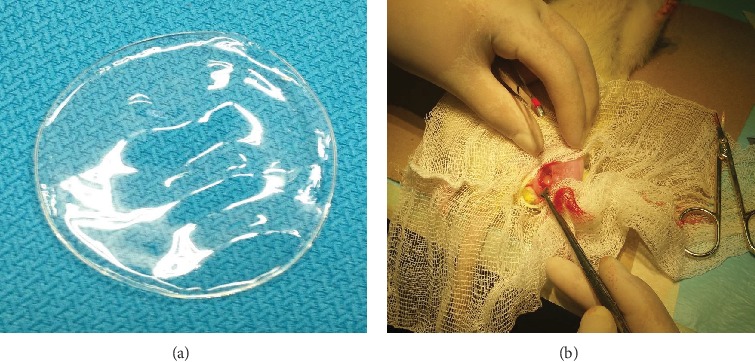
Surgical procedure on Wistar rats for *in vivo* assay. (a) Hydrogel film swollen in PBS used in surgery. (b) Surgical area in the hind leg of the rat before hydrogel film was placed.

A surgical incision was made in Wistar rats. Once the hind leg of each rat was cleaned and immobilized on the surgical table (Figures 1(a) and 1(b)), swelled and sterilized hydrogel samples was placed in the subdermal surgery section. A surgical incision was made in the control group, but no hydrogel was implanted. After surgery, the rats recovered for 2 h, and their weight, food intake, and health were monitored. Then, the rats were sacrificed under euthanasia by decapitation, after exposure to CO_2_ for 5 minutes in a chamber (Guidelines for the Care and Use of Laboratory Animals 062-200-1999) at 15, 30, 60, and 90 days. Inflammation in the surgical area was examined postmortem. A cross-section of the biopsy was fixed overnight in 4% paraformaldehyde and embedded in paraffin. Samples (3 *μ*m thick) were prepared for histological analysis [11]. Hematoxylin-eosin was used to examine the morphology of the tissue around the hydrogel film.

## 3. Results

### 3.1. Characterization of Hydrogels

Table 1 shows the results of the mechanical and humidity properties of LT hydrogel films. Tensile strength and elongation values showed to be gradually decreased according to the concentration of LT (58-46 N/mm^2^ and 25-15 mm, respectively), while the water content was increasing with the concentration of LT (31-36%). Interestingly, the angle contact values of LT hydrogel films demonstrated to have better hydrophilic properties when higher LT concentrations were used (50-36°). These results indicate that the presence and particular concentrations of LT intervene in the mechanical and hydrophilic properties of hydrogel films.

**Figure 2:**
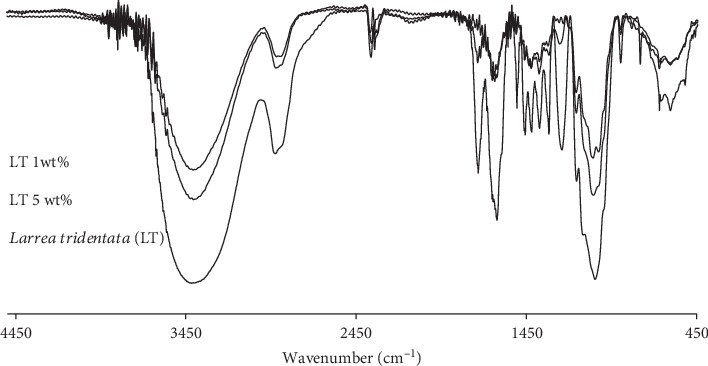
FT-IR spectra of hydrogel films in dry conditions of *Larrea tridentata* (LT) and cellulose films with different contents of LT.

**Figure 3:**
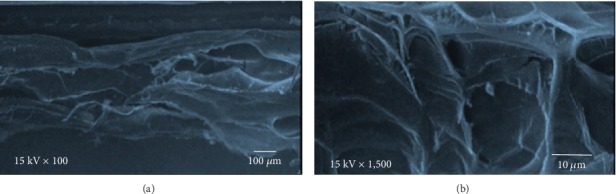
SEM images of the cellulose films' surface containing 5 wt% LT with different magnifications.

FT-IR measurements of LT cellulose films in dry conditions were carried out.  Figure 2 shows spectral regions related with the presence of cellulose and LT in the hydrogel samples. The frequencies around 680 to 920 cm^−1^ are associated with aromatic groups C=C, (C-H) of aromatic rings related to compounds contained in LT, along with frequencies around 1045 cm^−1^ assigned to vibrations to tension of C-O. The values of 1160 and 1240 cm^−1^ correspond to ester groups C-O, which coincided with the described peaks for LT components [23]. The frequencies of 1360 cm^−1^ are related to the deformation of C-O of the phenolic groups related to *Larrea tridentata*. Peaks around 2830 and 2872 cm^−1^ correspond to asymmetric vibrations of aliphatic CH_3_ groups reported in LT spectra. These peaks were also observed in cellulose films containing 1 and 5 wt% LT. Moreover, these spectral data presented broad peaks centered at 3400 cm^−1^ related to stretching vibrations of the bonded and nonbonded hydroxyl groups in cellulose and LT. Moreover, LT cellulose films were analyzed by SEM. As shown in Figures 3(a) and 3(b), the homogeneous sample was observed. No significant difference was detected in the surface of films containing different LT contents. This could suggest LT was well integrated in cellulose solution during the preparation of the several samples.

### 3.2. Protein Adsorption

The biocompatibility properties of the LT hydrogels were assessed by bicinchoninic assay with PBS and FBS (Table 2). The use of PBS media increased gradually the concentration of protein absorption according to the concentration of LT (25-33 *μ*g/cm^3^); however, the FBS media demonstrated to have opposite behavior than PBS, decreasing the values of protein absorption consistently to the LT concentration (19-11 *μ*g/cm^3^). These results suggest that the level of protein absorption could be directly related to the type of solution used.

### 3.3. Morphology of Adherent Cells

**Figure 4:**
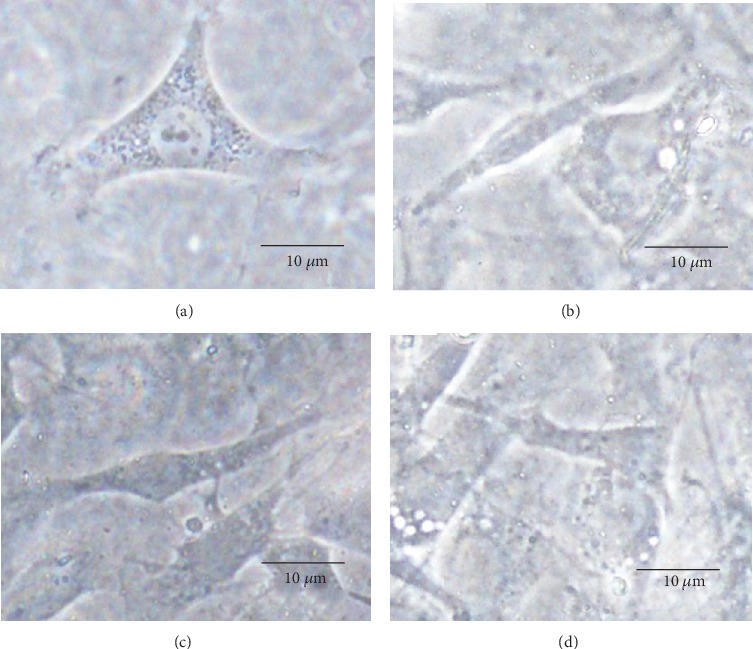
Phase-contrast light images of adherent cells on LT hydrogel films after 4 h in culture. (a) Adherent cells on the PS dish and cellulose hydrogel with (b) 1 wt% LT, (c) 3 wt% LT, and (d) 5 wt% LT.

Figure 4 shows phase-contrast images of adherent cells on the PS dish and hydrogel surface at various mimosa contents after 4 h in culture. The morphology of adherent cells differed between the PS dish and the LT hydrogels.  Figure 4(a) shows cells with a primarily rounded shape in the first several hours of culture. In contrast, Figures 4(a)–4(c) show adherent cells with diffuse borders and an anisotropic shape more similar to mature fibroblasts after 4 h in culture. The adherent cells had a long anisotropic shape at early time points.

**Figure 5:**
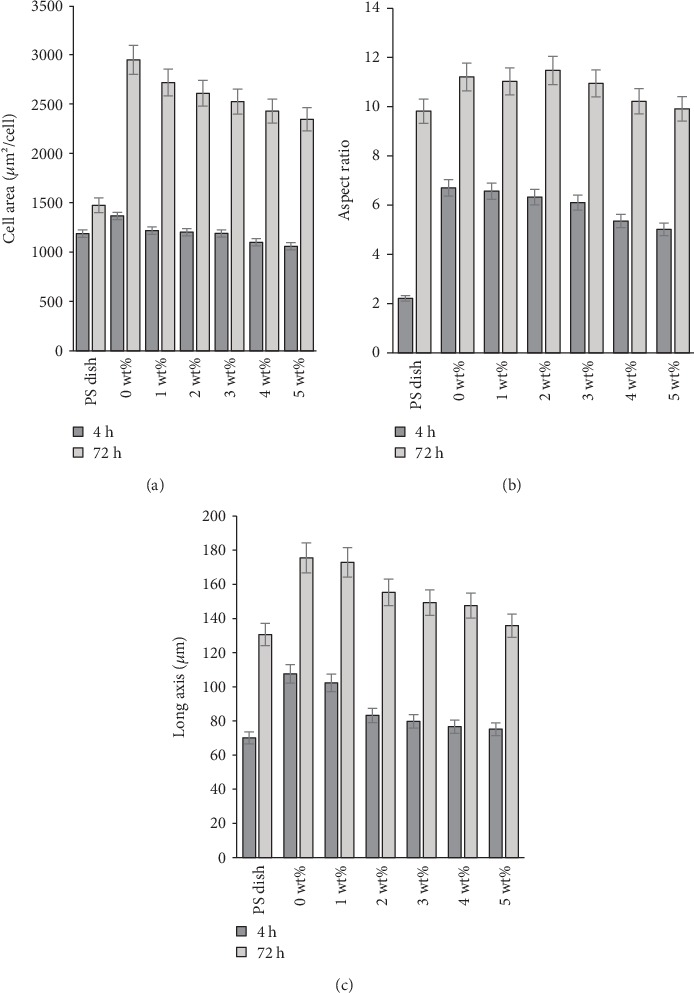
Morphological parameters of adherent cells at 4 and 72 h of cell culture. (a) Projected cell area, (b) aspect ratio, and (c) length of the long axis of cells. The PS dish was used as the control. Bars correspond to mean ± standard deviation for *n* = 10 for hydrogel surfaces prepared with various concentrations of LT.

Figure 5 describes the cell morphology parameters such as the aspect ratio, cell area, and long axis. The cell area, aspect ratio, and long axis parameters were predominately higher after 72 h compared to 4 h for the presence of LT hydrogel films observing significant diminishing concentrations of cells, cell length, and, in some cases, aspect ratio according to the LT concentration (*p* < 0.05). These results suggest that the variations from morphology characteristics of cells exposed to those hydrogel films could be strongly associated with the presence and concentration of LT as well as longer exposure times. In general, the LT hydrogel films at low concentrations showed to be a substrate that improved the morphology of cells when the time increased.

**Figure 6:**
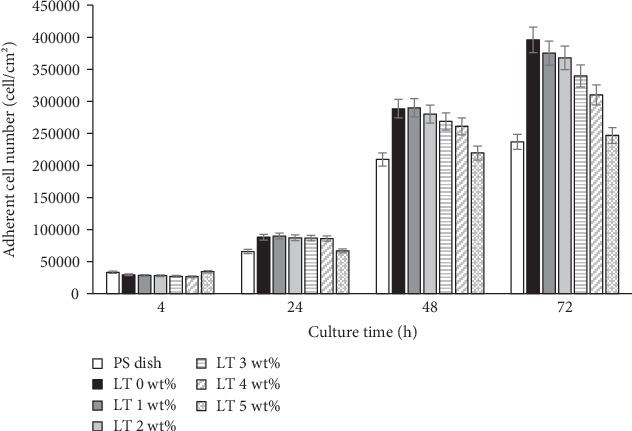
Adherent cell number on LT cellulose hydrogel films at various culture times. LT content was varied from 1 to 5 wt%. Values indicate statistically significant differences from the PS dish, used as the control (*p* < 0.05).

As shown in Figure 6, the number of adherent cells decreased slightly with higher LT levels. In the first 4 h of culture, there were more adherent cells on cellulose films without LT, whereas there were fewer on the PS dish. In the case of LT films, there was no significant difference. After 24 h in culture, the number of adherent cells was higher in all samples. There was no difference in adherent cell numbers on LT films. Moreover, the differences in adherent cell numbers were more evident in LT hydrogel films after 48 and 72 h of culture, decreasing at higher LT levels in the sample. In all cases, the PS dish had fewer adherent cells, and the highest number was seen on cellulose films without LT. Many adherent cells were observed in all LT films—higher than in the PS dish. These results were significant in all hydrogel samples (ANOVA, Student's *t*-test, *p* < 0.05, *n* = 6). That means that the hydrogel films with LT can gradually promote more cell adhesion with lower LT concentrations and longer periods of time.

### 3.4. *In Vivo* Assay

**Figure 7:**
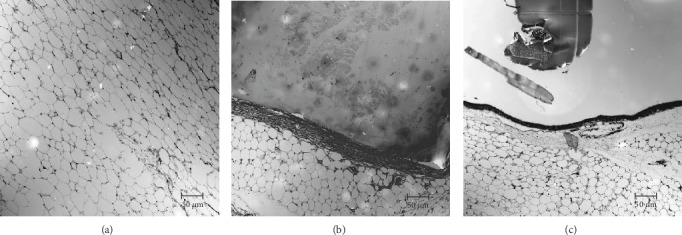
Confocal images of tissue formed in the surgical area using cellulose hydrogel film with 5 wt% LT. (a) Control, (b) 15 days after surgery, and (c) 90 days after surgery.

Confocal images of cellulose hydrogel film with 5 wt% LT in Wistar rats are shown in Figure 2. No evident inflammatory reaction was observed in the hind legs of the rats before euthanasia. Further, the rats were sacrificed at various times (15, 30, 60, and 90 days) for histological evaluation. At this time, no inflammatory reaction occurred in the surgical area postmortem in any rat. There was no evidence of an inflammatory reaction near the hydrogel film or in the surgical area in any sample. For example, Figure 7(b) shows a histological section of a hind leg 15 days after the surgery; the hydrogel portion did not undergo any significant shrinkage. There was no evidence of an inflammatory reaction near the hydrogel sample. Mature adipose tissue was noted in the sample material. The fibrous area next to the hydrogel film in Figure 7(b) might have been related to the healing process after surgery. On the other hand, Figure 7(c) shows the presence of epithelial tissue and a small portion of hydrogel material adjacent to the leg 90 days after surgery. The difference in the portion of the material was significant compared with Figure 7(b). Mature adipose tissue was also observed. There was no inflammatory reaction around the hydrogel sample. Compared with Figure 7(b), there was less fibrous tissue. There were no significant differences in the appearance of the tissue or the volume of the hydrogel sample for any LT hydrogel.

**Figure 8:**
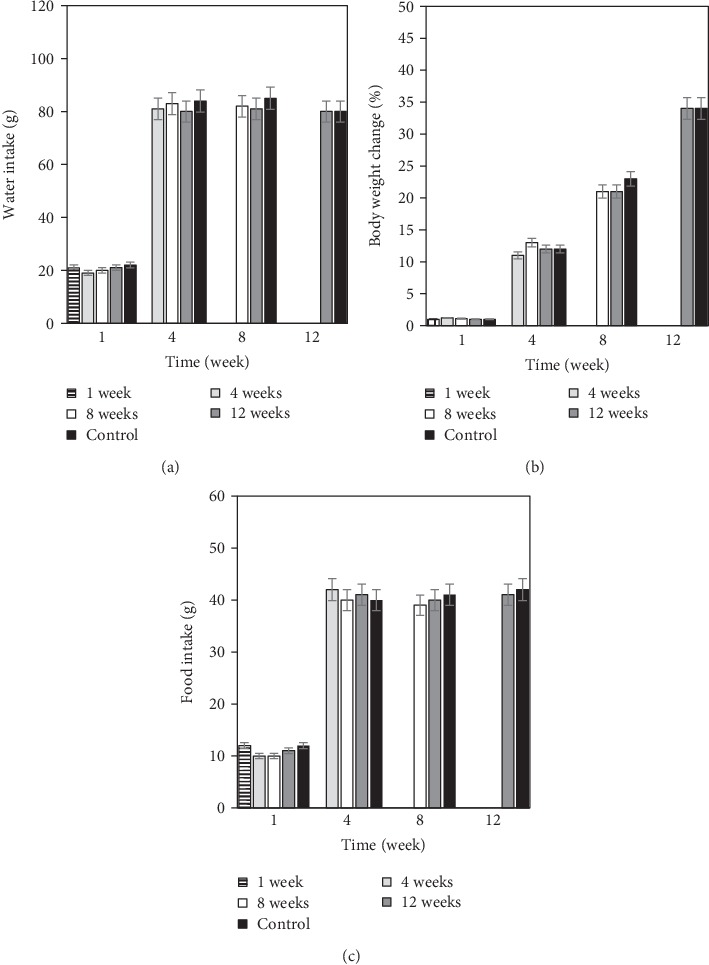
Water intake (a) and food intake (b) changes of rats after surgery. Each bar indicates the tested group for different periods. Body weight change (c) after implantation of LT cellulose film. Each bar indicates the group analyzed for different periods. Weight loss of more than 20% indicates toxicity. The mean value (±SEM) of obtained data was considered.

In addition, the implantation of LT cellulose films in Wistar rats did not cause any deaths in the tested specimens during the 12 weeks of *in vivo* assay.  Figure 8 shows changes in water intake and food intake as well as body weight change of the rats. No significant difference was observed in the values obtained in tested rats compared with the control group. Moreover, body weight of rats increases normally after surgery. This tendency was observed in all tested specimens. It has been reported that decreased body weight or variation around 10-20% is indicative of toxicity reaction.

## 4. Discussion

This study determined that cellulose hydrogel films from agave bagasse waste with different concentrations of LT demonstrated to have adequate mechanical, humidity, and good protein absorption characteristics as well as good cell and animal biocompatibility properties from *in vitro* and *in vivo* tests to be used with high potential for tissue regenerative applications in biomedical fields. Also, the presence and concentration of LT was associated with specific physical properties of hydrogel films demonstrating the best properties when the LT concentration was used at low doses (5%) in a long period of time (72 h). Furthermore, the subchronic expositions (90 days) of hydrogel films with LT from animal tests suggest the safe use with biocompatibility properties and morphology and cell adhesion assays support the application to be used as a cell platform or scaffold for tissue regenerative therapies.

With higher LT levels in the film, tensile strength declined, similar to our previous reports on hydroxyethyl cellulose [24]. This result could be attributed to the interference of LT with the chemical bonds between cellulose fibers and DMAc/LiCl in the solvent system. This tendency was also observed for elongation values, which decreased with higher LT concentration (Table 1), perhaps due to the interference of cellulose fibers with the DMAc/LiCl system [19]. As discussed, the presence and concentration of LT also affected tensile strength and elongation values. Also, the water content (i.e., swelling) of the hydrogel samples rose slightly as the LT content increased (Table 1), perhaps due to the decrease in strain at higher LT percentages, allowing more water into the cellulose scaffold. This tendency was also confirmed for the contact angle. As expected, the contact angle decreased at higher LT contents in the film, demonstrating a more hydrophilic surface on hydrogel films that were prepared with 5 wt% LT. On the other hand, protein adsorption is also important in cell adhesion and proliferation. A slight difference was observed in protein adsorption by the hydrogels with increased LT content. Nevertheless, FBS content decreased at higher LT levels in the film [20], perhaps due to the biocompatibility of LT—wherein greater stiffness does not affect the surface adsorption of PBS and FBS. The opposite patterns for PBS and FBS adsorption could be attributed to the surface properties of the hydrogel films. PBS prefers surfaces with hydrophilic properties, whereas FBS adsorbs more easily to hydrophilic surfaces [24, 25]. Furthermore, the morphology of adherent cells was similar in hydrogel films, despite the slightly negative effect at higher LT content. Lin and Helmke and Kidoaki and Matsuda [24, 26] observed the influence of mechanical properties on cell adhesion and mechanotaxis due to decreased stiffness of the film.

A primarily anisotropic shape was observed in all LT films, suggesting the advantages of our LT films compared with the PS dish, providing a more suitable surface for cells that are more similar in shape to mature fibroblasts, even in the first several hours of culture. Moreover, the morphology of adherent cells did not differ with higher LT content in the film, perhaps due to the biocompatibility of LT [10, 16]. Morphological parameters are related to the properties of materials and protein adsorption. Additionally, all parameters evaluated in this study showed a strong tendency to determine particular mechanical properties of hydrogel films with LT as well as variations in cell morphology aspects, cell viability, and *in vivo* reactions from animal models with respect to LT content in the hydrogel films. As LT content increased, the aspect ratio, cell area, and long axis fell slightly. This tendency could be also attributed to the effect of LT on the mechanical properties of the films. A more evident difference in the morphology parameters versus LT content was expected, but in contrast, a slight tendency was obtained in morphological variations from cells exposed to hydrogel films and content of LT. This could be attributed to the protein adsorption results. Protein adsorption has an impact on cell adhesion; moreover, changes in a material's surface can also affect cell spreading and cell morphology.

On the other hand, recent studies have prepared and tested various hydrogels using *in vivo* tests, basically rodents, which have suggested that those hydrogels containing peptide nanofibers [27], exosome-loaded alginate [28], gelatin microribbon scaffolds filled with degradable nanoporous chondroitin sulfate [29], tetra-PEG hydrogel-based aspirin [30], and other compounds into hydrogels [31–33] have the potential to be used for biomedical applications, principally for bone or tissue regeneration. Our results from the *in vivo* test indicated that the presence of cellulose hydrogel films with 5 wt% LT in Wistar rats showed histologically no evident inflammatory reaction when the hydrogels were exposed during 15, 30, 60, and 90 days. It is very well known that *L. tridentata* possesses various benefits for medicine due to its excellent antimicrobial, antioxidant, and antitumor properties as well as great biocompatibility [12–14]; however, other evaluations should be performed to determine particularly the clinical use for the biomedical field. Moreover, water intake and food intake were registered during the *in vivo* assay and compared with feeding behavior of the control group and no significant difference was observed. In addition, body weight change was also calculated. Body weight of rats did not decrease during the tested period of time after surgery. In all the specimens, body weight showed a normal body weight increase during the assay. Therefore, these data indicated that LT cellulose films exhibited no toxicity of inflammatory reaction over a period of 12 weeks.

Additionally, for further application of LT cellulose films in medicine, fibroblast adhesion to the hydrogel surface should be considered. Although this study obtained acceptable physical and biological properties of hydrogel films from agave bagasse using different concentrations of LT, it is still needed to evaluate this behavior using other parameters, instruments, and particular biologic conditions. Microscope images of adherent fibroblasts on the hydrogel films should be used to calculate cell density; but also, other *in vitro* evaluations using different cell lines as well as chemical and clinical evaluations in animals such as clinical and hematological biomarkers, bioabsorption, systemic distribution, and regenerative tests in conjunctive, epithelial, or bone tissues should also be developed to indicate the safe use of this hydrogel film with LT for possible biomedical applications.

## 5. Conclusion

We have performed an evaluation of the cytocompatibility and biocompatibility of LT cellulose hydrogel films. Cytocompatibility and biocompatibility properties were not affected by the addition of LT. The cell adhesion number, cell area, aspect ratio, and long axis did not differ for adherent cells between LT contents. The present work emphasizes the addition of LT to hydrogel films to obtain soft materials with good cell adhesion and expand the application of cellulose film in biomedicine to be used as regenerative biomaterial where platforms or scaffolds are required.

## Figures and Tables

**Table 1:** Properties of cellulose hydrogel films with LT.

LT content (%)	Tensile strength (N/mm^2^)	Elongation (mm)	Water content (%)	Contact angle (°)
0	58 ± 0.21	25 ± 0.19	31 ± 0.22	50 ± 0.1
1	56 ± 0.57	24 ± 0.61	31 ± 0.15	47 ± 0.3
2	53 ± 0.24	22 ± 0.27	32 ± 0.27	43 ± 0.2
3	51 ± 0.33	21 ± 0.42	33 ± 0.16	39 ± 0.1
4	49 ± 0.26	18 ± 0.41	35 ± 0.11	37 ± 0.1
5	46 ± 0.51	15 ± 0.39	36 ± 0.23	36 ± 0.3

Each column represents the mean (±SEM) number of samples tested at 25°C to obtain a reliable value. Mean ± SEM for *n* = 5 for each test (*p* < 0.05).

**Table 2:** Protein adsorption on films and evaluation of biocompatibility properties.

LT content (%)	PBS (*μ*g/cm^3^)	FBS (*μ*g/cm^3^)
0	25 ± 0.16	19 ± 0.21
1	26 ± 0.24	18 ± 0.13
2	27 ± 0.11	16 ± 0.25
3	29 ± 0.19	15 ± 0.23
4	30 ± 0.22	13 ± 0.18
5	33 ± 0.11	11 ± 0.21

Each column represents the mean (±SEM) number of samples tested at 25°C to obtain a reliable value. Mean ± SEM for *n* = 6 for each test (*p* < 0.05).

## Data Availability

All data obtained from this study can be found in the research archives of the Master's Program in Dental Sciences of the Autonomous University of Ciudad Juarez and can be requested through the corresponding author.
